# Rural-urban differences in post-9/11 women veterans’ firearm ownership and characteristics

**DOI:** 10.1186/s40621-025-00622-9

**Published:** 2025-11-03

**Authors:** Ashley M. Griffith, Christin Miller, Nicole M. Caulfield, Jeri E. Forster, Ryan Holliday, Claire A. Hoffmire, Joseph A. Simonetti, Talia L. Spark, Alexandra L. Schneider, Lindsey L. Monteith

**Affiliations:** 1 VA Rocky Mountain Mental Illness Research, Education and Clinical Center (MIRECC) for Suicide Prevention, Aurora, CO USA; 2 Department of Physical Medicine and Rehabilitation, University of Colorado Anschutz Medical Campus, Aurora, CO USA; 3 Firearm Injury Prevention Initiative, University of Colorado Anschutz School of Medicine, Aurora, CO USA; 4 VISN 2 Center of Excellence for Suicide Prevention, Canandaigua, NY USA; 5 Spark M. Matsunaga VA Medical Center, VA Pacific Islands Healthcare System, Honolulu, HI USA; 6 Department of Psychiatry, University of Colorado Anschutz Medical Campus, Aurora, CO USA; 7 Division of Hospital Medicine, University of Colorado Anschutz Medical Campus, Aurora, CO USA; 8 Behavioral Health and Health Policy Practice, Westat, Rockville, MD USA

**Keywords:** Women, Veterans, Rurality, Firearms, Suicide prevention

## Abstract

**Background:**

Access to firearms is associated with elevated risk for suicide; however, knowledge of firearm ownership and characteristics among rural residing women Veterans remains limited. Given increasing rates of firearm suicide among women Veterans, we examined if rurality was associated with firearm ownership and firearm characteristics (type, number, reasons for ownership, perceived safety, storage) among women Veterans.

**Methods:**

In 2020, 525 post-9/11 era women Veterans completed a survey assessing firearm ownership and characteristics. Prevalence ratios (PR) and 95% confidence intervals (CI) were estimated to compare differences in firearm ownership and related characteristics between rural and urban residing women Veterans, using Poisson regression with robust standard errors.

**Results:**

Rural, relative to urban, residing women Veterans were significantly more likely to report personal firearm ownership (PR = 1.30; 95%CI: 1.07, 1.57) and household firearm ownership (PR = 1.22; 95%CI: 1.05, 1.41). Significant differences were not detected regarding types of firearms, number of firearms, firearm storage methods, reasons for owning, or perceived sense of safety from the presence of a firearm in the home. In a post-hoc sensitivity analysis with an alternative conceptualization of rurality, rural residing, compared to urban residing, women Veterans were more likely to own only a long gun or both long guns and handguns.

**Conclusions:**

Assessing for personal ownership and household firearm access is an important component of suicide prevention for both rural and urban residing women Veterans and may be especially relevant to post-9/11 women Veterans residing in rural areas.

**Supplementary Information:**

The online version contains supplementary material available at 10.1186/s40621-025-00622-9.

## Background

In 2022, female^1^ Veterans had an age-adjusted suicide rate (14.2/100,000) 92.4% higher than non-Veteran adult females (7.4/100,000) in the United States (U.S.), a rate that rose 55.4% from 2001 to 2022 [1]. Additionally, firearms are the leading method of suicide among female Veterans [1]. The rate of firearm suicides was also 144.4% higher among female Veterans, compared to non-Veteran U.S. adult females, with 45.4% of suicide deaths among female Veterans involving a firearm in 2022 [1].

The three-step theory (3ST) of suicidal behavior explains how suicidal ideation progresses to suicide attempt [2]. A key contributor is the capability for suicide, or habituation to pain and death, which includes practical capability – the access, knowledge, and skill to use lethal means – which is associated with risk for firearm suicide [2–5]. Firearm acceptability, such as social and cultural beliefs and norms (e.g., encouraging participation in firearm-related activities), also contributes to firearm availability and knowledge [4]. Moreover, firearm ownership and household access are independently associated with risk for suicide [6, 7], including firearm suicide [8], regardless of the presence of mental health disorders [9].

Despite increasing rates of firearm suicide among women Veterans [1], until recently, most research examining firearm behaviors and characteristics among U.S. Veterans focused on samples predominantly comprised of men or did not stratify by gender. This has limited the understanding of firearm behaviors and associated characteristics and how to prevent firearm suicide among women Veterans [10]. Nonetheless, initial findings have highlighted the importance of furthering this research. Compared to women non-Veterans, women Veterans are more likely to own any firearms and multiple types of firearms (e.g., handguns, rifles) [11], with 22.2% reporting firearm behaviors (e.g., making household firearms more accessible, purchasing a firearm or ammunition) during the early part of the coronavirus disease 2019 (COVID-19) pandemic [12]. Further, among post-9/11 women Veterans using Veterans Health Administration (VHA) reproductive healthcare services, 54.0% reported firearm access; of those, 38.0% reported personal ownership and 38.9% reported that someone else in their household owned a firearm [13]. Relatedly, women Veterans are more likely than men Veterans to live in a household with a firearm they do not own. This finding may help with understanding the higher proportion of firearm suicide deaths through firearms owned by others (e.g., spouse/partner, parent, friend) among women, relative to men [14]. Additionally, 34.3% of male and 13.2% of female Veteran personal firearm owners reported storing at least one firearm loaded and unlocked [15]. In a sample of post-9/11 women Veterans using VHA reproductive healthcare services, of those with firearm(s) access, 17.8% reported all firearms were stored loaded and 21.9% reported all firearms were stored unlocked; furthermore, women with lifetime or past-month suicidal ideation were more likely to store their firearms loaded [13].

In addition to gender differences in firearm access and secure storage, women Veterans report nuanced factors driving their firearm access. Women Veterans often cite desire for personal safety, feeling secure, and protection as key reasons for firearm ownership and unsecure storage behaviors (i.e., loaded, unlocked), especially after experiencing interpersonal violence (e.g., sexual harassment, sexual assault) or perceived threats during deployment [12, 16–19]. These experiences may contribute to firearm storage behaviors that increase risk for suicide, as owning a firearm for protection has been associated with storing firearms unlocked and loaded [15], which may be associated with increased suicide risk [7, 20]. As firearms lethal means safety is a key component of suicide prevention, it is imperative to identify subgroups of women Veterans who are more likely to have access to firearms and unsecure firearm storage behaviors, as well as factors which may their influence firearm access and behaviors.

Geography (defined herein as the physical and contextual environment that individuals reside within, such as rural and urban areas [21]) has been noted to play a role in exposure to firearms [4], as well as firearm suicide [22]. Among U.S. Veterans, firearm owners are more likely to live in rural areas [23]. Veterans residing in rural, compared to urban, areas are less likely to store their firearms unloaded and locked [15]. In a Midwestern sample of women Veterans and service members from the Army and Air Force who had deployed, those living in rural areas were 1.6 times more likely to report having firearms or weapons nearby to feel secure [16]. Veterans residing in rural areas also face unique geographical stressors, including social and economic circumstances, isolation, and reduced access to evidence-based healthcare that may contribute to their heightened risk of suicide [24]. Indeed, among VA patients, Veterans from rural areas were 20–22% more likely to die by suicide than their urban counterparts in Fiscal Years (FY) 2003–2004 and 2007–2008, with 76.8% of these deaths attributed to a firearm injury in FY 2007–2008 [22]. While higher suicide rates for rural (versus urban) Veterans have been found for both male and female Veterans [25], sex differences have also been noted in the association between rurality and firearm suicide. Specifically, male Veterans from rural areas were significantly more likely to die from firearm suicide than urban-based male Veterans, whereas no significant differences were found in suicide methods between rural and urban female Veterans, among whom 45.2% and 35.1% of suicide deaths in FY 2007–2008, respectively, occurred via firearm injury [22]. However, as these data are nearly two decades old, and firearm suicide deaths have increased substantially among women Veterans [1], further research is necessary to understand if rurality is associated with differences in firearm access and characteristics among women Veterans. Such knowledge may help to inform suicide prevention strategies that are responsive to both gender-based and geographic considerations.

In sum, to our knowledge, despite associations between firearm access and unsecure storage with suicide, no studies have examined if rurality is associated with firearm ownership or firearm storage practices among women Veterans. In this secondary analysis, we examined the association between rurality and personal and household firearm ownership among post-9/11 women Veterans. We also examined associations between rurality and firearm characteristics, including number of firearms, firearm types, and storage practices. Based upon prior literature [16, 23], we hypothesized that women Veterans residing in rural, relative to urban, areas would have a greater likelihood of reporting personal firearm ownership, household firearm ownership (i.e., participant or anyone else they lived with currently owned a firearm), owning multiple (vs. 1) firearms, owning long guns or long guns and handguns as compared to only handguns, and engaging in high-risk storage practices (i.e., firearms stored unlocked and loaded). Additionally, we explored if, among firearm owners, there were differences between women Veterans in rural and urban areas regarding their primary reasons for firearm ownership, and if there were differences between women Veterans in rural and urban areas, and specifically firearm owners, and their perceived sense of safety associated with having firearms present in the home.

## Method

### Participants and procedures

Data for this secondary analysis were collected from 5/2020 to 12/2020 as part of a survey study designed to understand pre-, during, and post-deployment experiences associated with firearm access and storage practices among post-9/11 women Veterans [12, 19]. Eligible participants were women Veterans with at least one post-9/11 deployment, aged 18–89-years-old, and enrolled in the VHA. 3,000 women Veterans identified through the VA Corporate Data Warehouse (CDW) and VA-DoD Identity Repository (VADIR) were invited to participate. Data collection occurred online or via a mailed paper survey. To facilitate participation and forthright disclosure [26, 27], participants could participate anonymously or non-anonymously with a $20 incentive. This study was approved by the local Institutional Review Board and VA Research and Development Committee.

Most of the 3,000 mailed invitations sent were presumed delivered (i.e., not returned to sender; *n* = 2,606; 86.9%). Among delivered invitations, 576 individuals (22.1%) consented to participate, 528 of whom were eligible (92.0%). Individuals were excluded if they were currently serving on active duty or full-time National Guard or Reserves or if they had no history of deployment.

### Measures

#### Rurality

Participants self-identified whether they lived in a rural area by responding to the following question: “Which of the following best describes where you currently live?” with five response options: rural, small town, medium-sized town, suburb, and city. Due to the sample size, responses were dichotomized to reflect those who lived in a rural (rural, small town, medium-sized town) vs. urban setting (suburb or city) based upon general definitions of town size (e.g., medium towns usually have a population less than 20,000) and the Health Resources & Services Administration definition of rural as areas with less than 50,000 individuals [28– 29]. However, given that response options were not predefined for participants, an alternate conceptualization of rurality was also examined in a post-hoc sensitivity analyses in which medium-sized towns were categorized as urban (no changes were made to the categorization of the other response categories).

#### Demographics and military service

Participants’ demographic characteristics and military service histories were assessed for descriptive and confounder adjustment purposes.

#### Firearms and firearm characteristics

Participants were asked if they had ever personally owned a firearm (currently, previously, never) and if anyone they lived with currently owned firearm(s) (yes, no). These were combined to determine current household firearm ownership, operationalized as current personal ownership and/or ownership by someone living with the participant, regardless of where firearm(s) were stored. Participants were also asked the extent to which they would feel safer having a gun in the home, with responses dichotomized as yes (much, somewhat, or a little safer) or no (neutral or a little less, somewhat less, or much less safe). Current personal firearm owners were asked how many firearms they owned (*M* = 2.5, median = 2, range 1–40), which was collapsed into 1 vs. multiple. Current personal firearm owners were also asked about the types of firearms owned (handgun vs. long gun only or handgun and long guns) and their primary reason for owning firearms. While participants were asked to select their *primary* reason for owning a firearm, many who participated by paper selected multiple answer choices. All responses, whether singular or multiple, were included in analyses and recategorized into four categories: recreation (collection, hunting, other sporting use), protection (against strangers, people I know, animals), both, and other. Finally, personal and household firearm owners reported how firearms were stored. Firearm storage was categorized into a priori safety risk categories of low (locked and unloaded), intermediate (either unloaded and unlocked or loaded and locked), and high (loaded with ammunition and unlocked).

### Analyses

Descriptive statistics for demographics, military service, firearm ownership, and firearm characteristics were calculated for the overall sample and urban and rural subsamples. Potential confounders were considered a priori based on prior literature and theory and included marital status, sexual orientation, children in the home, race, ethnicity, age, education, and combat zone deployment; chi-square, t-tests, and Fisher’s exact tests, as appropriate, were conducted to compare characteristics between rural and urban women and to determine significant relationships with rurality (*p* <.05). Variables deemed not on the causal pathway and significantly associated with rurality, based on either definition of rurality (education, sexual orientation, and race), were included as covariates in adjusted models. Given that outcomes of interest were common (>10%), crude and adjusted modified Poisson regressions with robust error variance [30] were used to estimate prevalence ratios (PR) with 95% confidence intervals (CIs). This method was applied to examine the relationship between rurality and (1) current personal firearm ownership, (2) household firearm ownership, and (3) perceived safety from having a firearm in the home in the overall sample. In the subsample of current personal firearm owners, this method was applied to examine the relationship between rurality and (1) number of firearms owned (1 vs. more than 1), (2) type of firearms owned (handguns only vs. long guns only or both types), and (3) perceived safety from having a firearm in the home. Due to sample sizes limiting modeling ability, exploratory chi-square analyses were conducted among current personal firearm owners to examine the relationship between rurality and primary reasons for owning a firearm. Finally, among both household firearm owners and personal firearm owners, crude and adjusted modified Poisson regressions with robust error variance were used to examine the relationships between rurality and firearm storage risk (low/intermediate vs. high, and low vs. intermediate/high). All outcome variables were dichotomous. Analyses were conducted using SAS v9.4.

## Results

### Demographic and military service characteristics

In the overall sample, 54.48% lived in an urban area, while 45.52% lived in a rural area (see Table 1 for sample characteristics). The majority of participants were White (68.00%), non-Hispanic (85.38%), and heterosexual (86.42%). Most had served in the Army (52.57%) and reported combat zone deployment(s) (87.19%). Rural residing women were less likely to be Black, more likely to be heterosexual, and reported lower educational status than urban residing women (see Table 1 and Supplemental Table 1).

**Table 1:** Participant demographics and military service characteristics

Characteristic	Overall sample (*N* = 525)	Urban (*n* = 286, 54.48%)	Rural (*n* = 239, 45.52%)	T-test or chi-square *p*-values
	*M* (SD) or *n* (%)			
Demographics				
Age (*n* = 517)	42.59 (10.31)	42.38 (9.97)	42.85 (10.73)	0.24
*Race*^a^ *(*n *= 521)*				
White	357 (68.00%)	185 (64.69%)	172 (71.97%)	0.07
Black	121 (23.05%)	78 (27.27%)	43 (17.99%)	0.01
Native American/Alaskan Native	15 (2.86%)	6 (2.10%)	9 (3.77%)	0.25
Asian/Pacific Islander	23 (4.38%)	17 (5.94%)	6 (2.51%)	0.06
Additional Races	26 (4.95%)	12 (4.20%)	14 (5.86%)	0.38
*Ethnicity (*n *= 523)*				0.10
Hispanic	76 (14.62%)	48 (16.96%)	28 (11.81%)	
Non-Hispanic	444 (85.38%)	235 (83.04%)	209 (88.19%)	
*Sexual Orientation*^b^ *(*n *= 523)*				0.15
Heterosexual/straight	452 (86.42%)	239 (83.86%)	213 (89.50%)	
Gay/lesbian	41 (7.84%)	29 (10.18%)	12 (5.04%)	
Bisexual	26 (4.97%)	15 (5.26%)	11 (4.62%)	
Additional sexual orientations^c^	4 (0.76%)	2 (0.70%)	2 (0.84%)	
*Education Status (*n *= 524)*				0.008
High School or GED	12 (2.29%)	5 (1.75%)	7 (2.94%)	
Some college	85 (16.22%)	34 (11.89%)	51 (21.43%)	
Associate degree	92 (17.56%)	49 (17.13%)	43 (18.07%)	
Bachelor’s degree	184 (35.11%)	101 (35.31%)	83 (34.87%)	
Graduate or professional degree	151 (28.82%)	97 (33.92%)	54 (22.69%)	
*Current Partner Status (*n *= 524)*				0.91
Married/living together	303 (57.82%)	166 (58.04%)	137 (57.56%)	
Single/widowed/divorced/ separated	221 (42.18%)	120 (41.96%)	101 (42.44%)	
*Minors in the home*	246 (46.86%)	133 (46.50%)	113 (47.28%)	0.86
*Current Living Situation* ^b^ *(*n *= 524)*				0.68
Rent/own	492 (93.89%)	268 (93.71%)	224 (94.12%)	
Living with a relative/friend	29 (5.50%)	17 (5.94%)	12 (5.04%)	
Currently homeless	3 (0.57%)	1 (0.35%)	2 (0.84%)	
*Employment Status (*n *= 524)*				0.11
Currently employed	343 (65.46%)	191 (67.02%)	152 (63.60%)	
Unemployed, seeking employment	38 (7.25%)	26 (9.12%)	12 (5.02%)	
Unemployed, not seeking employment	61 (11.64%)	29 (10.18%)	32 (13.39%)	
Retired	82 (15.65%)	39 (13.68%)	43 (17.99%)	
Military Service Characteristics				
*Branch*^a^ *(*n *= 524)*				
Army	276 (52.57%)	151 (52.80%)	125 (52.30%)	0.91
Air Force	121 (23.05%)	71 (24.83%)	50 (20.92%)	0.29
Marine Corps	35 (6.67%)	15 (5.24%)	20 (8.37%)	0.15
Navy/Coast Guard	115 (21.90%)	60 (20.98%)	55 (23.01%)	0.57
*Rank at Separation* ^b^				0.18
Junior enlisted	359 (68.38%)	194 (67.83%)	165 (69.04%)	
Non-Commissioned Officer	75 (14.29%)	35 (12.24%)	40 (16.74%)	
Warrant Officer	6 (1.14%)	3 (1.05%)	3 (1.26%)	
Commissioned Officer	85 (16.19%)	54 (18.88%)	31 (12.97%)	
*Post-9/11 military service* * only (*n = *522*)	327 (62.64%)	175 (61.40%)	152 (64.14%)	0.52
*Number of Deployments*				0.70
One	253 (48.19%)	140 (48.95%)	113 (47.28%)	
Two or more	272 (51.81%)	146 (51.05%)	126 (52.72%)	
*History of Combat Zone* * Deployment (*n *= 523)*	456 (87.19%)	245 (85.66%)	211 (89.03%)	0.25

Urban = suburb, city. Rural = medium-sized town, small town, rural. N values for each characteristic represent the total number of respondents who provided a response to that item and may be fewer than the total sample included. T-test or chi-square tests conducted to examine differences between urban and rural women Veterans

^a^Not mutually exclusive

^b^Fisher’s Exact Test

^c^Includes asexual

### Firearm descriptive statistics

#### Ownership

Significantly more rural residing women Veterans reported current personal firearm ownership (50.85%) and household firearm ownership (65.11%), relative to urban residing women Veterans (39.72% and 53.02%, respectively; Table 2). Additionally, in the overall sample, 71.24% of rural residing women Veterans and 64.87% of urban residing women Veterans reported feeling safer with a firearm in the home, although a statistically significant difference was not observed. Similar results were found in the post-hoc sensitivity analysis with the alternate conceptualization of rurality (see Supplemental Table 2).

**Table 2:** Participant firearm ownership and characteristics by rurality

Characteristic	Overall sample (*N* = 525)	Urban (*n* = 286, 54.48%)	Rural (*n* = 239, 45.52%)	T-test or chi-square *p*-values
	*n* (%)			
Firearm Ownership/Safety				
* Current Personal Firearm* * Ownership (*n *= 518)*	232 (44.79%)	112 (39.72%)	120 (50.85%)	0.01
* Household Firearm* * Ownership (*n *= 516)*	302 (58.53%)	149 (53.02%)	153 (65.11%)	0.006
* Feel Safer with Firearm in* * Home (*n *= 512)*	347 (67.77%)	181 (64.87%)	166 (71.24%)	0.12
Firearm Characteristics				
Among Current Firearm Owners				
* Number of Firearms* * Owned (*n *= 221)*				0.10
One	102 (46.15%)	56 (51.85%)	46 (40.71%)	
Multiple	119 (53.85%)	52 (48.15%)	67 (59.29%)	
*Types of Firearms (*n *= 228)*				0.12
Handguns only	133 (58.33%)	70 (63.64%)	63 (53.39%)	
Long guns only or both	95 (41.67%)	40 (36.36%)	55 (46.61%)	
*Primary Reasons for Firearm* *Ownership*^a^ *(*n *= 232)*				0.09
Recreation	38 (16.38%)	15 (13.39%)	23 (19.17%)	
Protection	160 (68.97%)	86 (76.79%)	74 (61.67%)	
Recreation and protection	27 (11.64%)	9 (8.04%)	18 (15.00%)	
Other	7 (3.02%)	2 (1.79%)	5 (4.17%)	
*Feel Safer with Firearm in* *Home (*n *= 231)*	202 (87.45%)	95 (84.82%)	107 (89.92%)	0.24
*Firearm Storage Practices* ^b^ (*n* = 225)				0.37
Low risk	75 (33.19%)	41 (37.96%)	34 (28.81%)	
Intermediate risk	80 (35.40%)	36 (33.33%)	44 (37.29%)	
High risk	70 (31.42%)	31 (28.70%)	39 (33.33%)	
Among Household Firearm Owners				
*Firearm Storage Practices* ^b^ *(*n *= 295)*				0.28
Low risk	103 (34.92%)	57 (39.31%)	46 (30.67%)	
Intermediate risk	109 (36.95%)	51 (35.17%)	58 (38.67%)	
High risk	83 (28.14%)	37 (25.52%)	46 (30.67%)	

Urban = suburb, city. Rural = medium-sized town, small town, rural. N values for each characteristic represent the total number of respondents who provided a response to that item and may be fewer than the total sample included. T-test or chi-square conducted to examine differences between urban and rural women Veterans

^a^Fisher’s Exact Test

^b^Low risk was operationalized as locked and unloaded; intermediate risk as unlocked and unloaded or locked and loaded; and high risk as loaded and unlocked

#### Firearm characteristics

Among current firearm owners, 59.29% of rural residing and 48.15% of urban residing individuals reported owning multiple firearms (Table 2). Over half (53.39%) of rural residing women Veterans reported owning handguns only, compared to 63.64% of urban residing women Veterans. Most participants in both rural (61.67%) and urban (76.79%) areas endorsed protection as their primary reason for firearm ownership^2^. Additionally, most current firearm owners residing in rural (89.92%) and urban (84.82%) settings endorsed feeling safer with a firearm present in the home. Despite the magnitude of these differences, none of these differences were statistically significant. Among women Veterans who reported household firearm ownership, 30.67% in rural areas and 25.52% in urban areas reported high-risk firearm storage; 30.67% of rural and 39.31% of urban residing women Veterans reported low-risk firearm storage; significant differences were not detected^3^.

Post-hoc sensitivity analyses with the alternate conceptualization of rurality resulted in similar outcomes for most variables (see Supplemental Table 2). The primary difference was regarding firearm types: in sensitivity analyses, urban residing women Veterans (64.54%) were more likely to own handguns only than rural residing women Veterans (48.28%), whereas rural residing women Veterans (51.72%) were more likely to own long guns only or both long guns and handguns, compared to urban residing women Veterans (35.46%).

### Comparisons

Rurality was significantly associated with both current personal and household firearm ownership in both unadjusted and adjusted models (Table 3). Adjusting for education, race, and sexual orientation, rural residing women Veterans had 1.30 times (95%CI [1.07, 1.57]) higher prevalence of current personal firearm ownership and 1.21 times (95%CI [1.04, 1.40]) higher prevalence of household firearm ownership, compared to urban residing women Veterans. Significant associations were not detected between rurality with perceived sense of safety from having a firearm in the home (full sample; current firearm owners), number of firearms (current firearm owners), types of firearms (current firearm owners), or firearm storage (current firearm owners; household firearm owners).

**Table 3:** Unadjusted and adjusted Poisson regression models for firearm ownership and characteristics by rurality

Model	Unadjusted models				Adjusted models^a^	
	*n*	PR (95% CI)	*p*	*n*	PR (95% CI)	*p*
**Full Sample (*N*** **= 525)**						
Current personal firearm ownership (current ownership vs. no current/past ownership)	518	1.28 (1.06, 1.55)	0.01	516	1.30 (1.07, 1.57)	0.009
Household firearm ownership	516	1.23 (1.06, 1.42)	0.005	515	1.21 (1.04, 1.40)	0.01
Perceived sense of safety with firearm in home	512	1.10 (0.98, 1.24)	0.12	509	1.07 (0.95, 1.21)	0.28
**Current Personal Firearm Owners (*n*** **= 232)**						
Number of firearms (1 vs. multiple)	221	1.23 (0.96, 1.58)	0.10	221	1.26 (0.98, 1.61)	0.07
Types of firearms (handguns vs. long guns/both)	228	1.28 (0.94, 1.75)	0.12	227	1.26 (0.92, 1.73)	0.15
Perceived sense of safety with firearm in home (yes/no)	231	1.06 (0.96, 1.17)	0.25	230	1.04 (0.95, 1.15)	0.39
Firearm storage risk^b^ (low/intermediate vs. high)	225	1.16 (0.78, 1.72)	0.46	224	1.17 (0.79, 1.74)	0.43
Firearm storage risk^b^ (low vs. intermediate/high)	225	1.14 (0.95, 1.38)	0.16	224	1.13 (0.93, 1.37)	0.21
**Household Firearm Owners (*n*** **= 302)**						
Firearm storage risk^b^ (low/intermediate vs. high)	295	1.20 (0.83, 1.74)	0.33	294	1.23 (0.85, 1.77)	0.28
Firearm storage risk^b^ (low vs. intermediate/high)	295	1.14 (0.97, 1.35)	0.12	294	1.14 (0.96, 1.36)	0.13

PR = prevalence ratio; CI = confidence interval. N values for each characteristic represent the total number of respondents who provided a response to that item and thus included in that analysis. Urban = suburb, city. Rural = medium-sized town, small town, rural. Reference group = urban residing women Veterans. The independent variable was rurality (rural vs. urban); dependent variables (outcomes) are listed in the lefthand column

^a^Adjusted for education, race (Black vs. all other races), and sexual orientation (heterosexual vs. all other)

^b^Low risk was operationalized as locked and unloaded; intermediate risk as unlocked and unloaded or locked and loaded; and high risk as loaded and unlocked

The post-hoc sensitivity analyses with the alternate conceptualization of rurality yielded largely similar results, with rurality significantly associated with both current personal and household firearm ownership in both unadjusted and adjusted models (Supplemental Table 3). The primary difference observed in the sensitivity analysis was that, in both unadjusted and adjusted models, rural residing women Veterans had a significantly higher prevalence of owning just long guns or both long guns and handguns than urban residing women Veterans.

## Discussion

This secondary analysis sought to understand the association between rurality with firearm ownership and firearm characteristics among women Veterans. As hypothesized and consistent with prior research [16, 23], rural residing women Veterans had a higher prevalence of current personal and household firearm ownership, compared to urban residing women Veterans. Social and cultural beliefs and norms may contribute to these differences. U.S. adult firearm owners from rural areas are more likely to have grown up with household firearms, to have acquired a firearm before age 18, and to own firearms for hunting [31]. The higher prevalence of personal and household firearm ownership among rural women Veterans may contribute to the higher rates of suicide among rural female Veterans [25], given that both firearm ownership and household firearm access are associated with elevated suicide risk [6, 7].

Despite the magnitude of differences in various firearm characteristics between rural and urban Veterans, significant differences generally were not detected in the types or number of firearms owned. However, in a post-hoc sensitivity analysis with an alternative dichotomization of rurality (with medium-sized towns conceptualized as urban), urban residing women Veterans were more likely to solely own handguns than rural residing women Veterans, which aligns with prior research [11]. As such, these differences may reflect variations in rurality definitions and grouping, as well as how participants defined their own living environment. Of note, Veterans living in urban areas have also previously been found to be more likely to solely own a handgun [11]. Further, in prior research, 67.0% of women Veteran firearm owners reported owning multiple types of firearms [11], whereas within our sample, among current personal firearm owners, 41.7% owned both long guns and handguns or long guns only. Thus, there also may be variations in firearm characteristics among different cohorts of Veterans.

Furthermore, while there were not statistically significant differences in this sample between rural and urban residing women Veterans who currently owned a firearm regarding their primary reason for owning firearms or perceived sense of safety from having firearms, protection was, by far, the most common primary reason for ownership. Additionally, the vast majority of both rural and urban residing women Veterans reported feeling safer with a firearm in their home, whether specific to current firearm owners or examining the full sample. This aligns with prior work that has identified personal safety, especially following interpersonal violence, as a primary reason for firearm access among women Veterans [12, 16–18].

Past research has found urban residing Veterans to be more likely to engage in both low-risk and high-risk firearm storage practices, compared to rural residing Veterans, who are more likely to engage in intermediate-risk storage practices [15]. Overall, the directionality of our results—though not statistically significant—mapped onto these past results for both low- and intermediate-risk storage practices, with the opposite finding for high-risk storage (i.e., a higher percentage of rural residing women Veterans engaging in high-risk storage). Together, our findings suggest that assessing for both personal and household firearm access — risk factors for suicide — is particularly important when working with rural post-9/11 women Veterans. Additionally, the relatively high prevalence of personal and household firearm ownership and intermediate and high-risk firearm storage practices within our sample underscore the need to discuss secure firearm storage practices with women Veterans, irrespective of their rurality or urbanicity status. Unfortunately, only a minority of U.S. adults (8.2% of women) with household firearms have discussed firearms with a healthcare provider [32], and women are less likely to be asked about firearms than men [33]. Additionally, of 590 Veterans receiving VA mental healthcare who were surveyed, only 27.5% reported previously discussing firearms with a clinician [34], despite Veterans expressing openness and recognizing the importance of firearm storage safety discussions, regardless of level of comfort [35, 36]. These findings underscore the need to better understand barriers and facilitators to discussing firearms with Veterans. Studies have begun to explore women Veterans’ preferences for how firearm lethal means safety can be approached clinically [18] and with their intimate partners [37]. However, such work did not include rurality as a specific component and there are differences in how firearms are viewed and used in rural communities [18]; consequently, an important next step for future research is to understand if rural and urban residing women Veterans differ in their preferences and experiences regarding firearm lethal means safety.

As results were based on a secondary analysis of data from a convenience sample of post-9/11 women Veterans enrolled in VHA care, an important consideration is generalizability. Weighting was not possible due to limited data on the population of post-9/11 women Veterans and anonymous participation. In addition, we were unable to explore differences between responders and non-responders due to anonymous participation. However, relative to the population of post-9/11 women Veterans [38], our sample appears to be similar in racial and ethnic composition, yet older and more highly educated.^4^ Due to the option to participate in the survey anonymously, we relied upon self-identified rurality and were unable to examine concordance between self-reported rurality and addresses for the full sample; respondent misclassification of rurality (e.g., self-defining a small town as a medium-sized town) is possible. Relatedly, while areas were grouped based upon definitions from the Health Resources & Services Administration and MeasuringHow [28, 29], important differences may exist between each of these geographical areas that could not be examined further. Finally, dichotomizing variables such as number of firearms owned (1 vs. multiple) limits a more nuanced understanding of whether rurality is associated with a higher number of firearms among women Veterans.

### Conclusions

To our knowledge, this is one of the first studies to examine if rurality is associated with firearm ownership and firearm characteristics among women Veterans, a population whose rate of firearm suicide has drastically increased over the past two decades. Findings indicate an elevated prevalence of both personal and household firearm ownership among rural, relative to urban, women Veterans. This has important implications for injury and suicide prevention in this population. Research to explore rural women Veterans’ experiences with, and preferences for, firearm lethal means safety strategies is needed to inform tailoring such initiatives to be responsive to rural women Veterans’ needs and contexts.

## Supplementary Information

Supplementary Material 1

## Data Availability

To access study materials, contact Lindsey Monteith, Ph.D. at lindsey.monteith@va.gov

## Footnotes

1 Throughout the manuscript, male and female are used in instances where results were based on sex assigned at birth, whereas women and men are used when results were based on gender.

2 Percentages were higher when considering those who endorsed responses consistent with both protection and recreation.

3 Similarly, when examining storage among the subset of women Veterans who personally owned firearms, significant differences also were not observed in firearm storage.

4 In 2018, approximately 23% of the post-9/11 women Veteran population were non-Hispanic, Black and 12% were Hispanic. Additionally, 78% were under the age of 45 years old. Lastly, approximately 45% of post-9/11 women Veterans had completed some college, with 41.1% having completed at least a Bachelor’s degree [38].
