# Supplementary material for: Rural-urban differences in post-9/11 women veterans’ firearm ownership and characteristics
Source: Inj Epidemiol. 2025 Nov 3;12:73. doi: 10.1186/s40621-025-00622-9 (PMC12581480; doi:10.1186/s40621-025-00622-9)
Supplement: Supplementary file 1 — Supplementary Material 1 [file 40621_2025_622_MOESM1_ESM.docx]

**Supplemental Table 1**

*Participant Demographics and Military Service Characteristics – Sensitivity Analyses* *with Alternate Conceptualization of Rurality*

|  | Urban  (*n*=355, 67.62%) | Rural  (*n*=170, 32.38%) | T-test or chi-square p-values |
| --- | --- | --- | --- |
| Characteristic | *M* (SD) or *n* (%) | |  |
| Demographics |  |  |  |
| Age (*n*=517) | 42.14 (9.85) | 43.56 (11.19) | 0.05 |
| *Race^a^* *(*n*=521)* |  |  |  |
| White | 238 (67.04%) | 119 (70.00%) | 0.50 |
| Black | 88 (24.79%) | 33 (19.41%) | 0.17 |
| Native American/Alaskan Native | 10 (2.82%) | 5 (2.94%) | 0.94 |
| Asian/Pacific Islander | 19 (5.35%) | 4 (2.35%) | 0.12 |
| Additional Races | 13 (3.66%) | 13 (7.65%) | 0.05 |
| *Ethnicity (*n*=523)* |  |  | 0.33 |
| Hispanic | 55 (15.67%) | 21 (12.43%) |  |
| Non-Hispanic | 296 (84.33%) | 148 (87.57%) |  |
| *Sexual Orientation^b^ (*n*=523)* |  |  | 0.008 |
| Heterosexual/straight | 296 (83.62%) | 156 (92.31%) |  |
| Gay/lesbian | 36 (10.17%) | 5 (2.96%) |  |
| Bisexual | 20 (5.65%) | 6 (3.55%) |  |
| Additional sexual orientations^c^ | 2 (0.56%) | 2 (1.18%) |  |
| *Education Status (*n*=524)* |  |  | 0.01 |
| High School or GED | 7 (1.98%) | 5 (2.94%) |  |
| Some college | 46 (12.99%) | 39 (22.94%) |  |
| Associate degree | 67 (18.93%) | 25 (14.71%) |  |
| Bachelor’s degree | 121 (34.18%) | 63 (37.06%) |  |
| Graduate or professional degree | 113 (31.92%) | 38 (22.35%) |  |
| *Current Partner Status (*n*=524)* |  |  | 0.61 |
| Married/living together | 208 (58.59%) | 95 (56.21%) |  |
| Single/widowed/divorced/separated | 147 (41.41%) | 74 (43.79%) |  |
| *Minors in the home* | 171 (48.17%) | 75 (44.12%) | 0.38 |
| *Current Living Situation*^b^ *(*n*=524)* |  |  | 0.37 |
| Rent/own | 332 (93.79%) | 160 (94.12%) |  |
| Living with a relative/friend | 21 (5.93%) | 8 (4.71%) |  |
| Currently homeless | 1 (0.28%) | 2 (1.18%) |  |
| *Employment Status (*n*=524)* |  |  | 0.09 |
| Currently employed | 238 (67.23%) | 105 (61.76%) |  |
| Unemployed, seeking employment | 30 (8.47%) | 8 (4.71%) |  |
| Unemployed, not seeking employment | 38 (10.73%) | 23 (13.53%) |  |
| Retired | 48 (13.56%) | 34 (20.00%) |  |
| Military Service Characteristics |  |  |  |
| *Branch^a^ (*n*=524)* |  |  |  |
| Army | 190 (53.52%) | 86 (50.59%) | 0.53 |
| Air Force | 81 (22.82%) | 40 (23.53%) | 0.86 |
| Marine Corps | 23 (6.48%) | 12 (7.06%) | 0.80 |
| Navy/Coast Guard | 74 (20.85%) | 41 (24.12%) | 0.40 |
| *Rank at Separation^b^* |  |  | 0.48 |
| Junior enlisted | 243 (68.45%) | 116 (68.24%) |  |
| Non-Commissioned Officer | 46 (12.96%) | 29 (17.06%) |  |
| Warrant Officer | 4 (1.13%) | 2 (1.18%) |  |
| Commissioned Officer | 62 (17.46%) | 23 (13.53%) |  |
| *Post-9/11 military service* *only (*n=*522*) | 223 (63.17%) | 104 (61.54%) | 0.72 |
| *Number of Deployments* |  |  | 0.06 |
| One | 181 (50.99%) | 72 (42.35%) |  |
| Two or more | 174 (49.01%) | 98 (57.65%) |  |
| *History of Combat Zone Deployment (*n*=523)* | 306 (86.20%) | 150 (89.29%) | 0.32 |

*Note.* Urban = medium-sized town, suburb, city. Rural = small town, rural. N values for each characteristic represent the total number of respondents who provided a response to that item and may be fewer than the total sample included. T-test or chi-square tests conducted to examine differences between urban and rural women Veterans.

^a^Not mutually exclusive.

^b^Fisher’s Exact Test.

^c^Includes asexual.

**Supplemental Table 2**

*Participant Firearm Ownership and Characteristics by Rurality* *– Sensitivity Analyses with Alternate Conceptualization of Rurality*

|  | Urban  (*n*=355, 67.62%) | Rural  (*n*=170, 32.38%) | T-test or chi-square p-values |
| --- | --- | --- | --- |
| Characteristic | *n* (%) | |  |
| Firearm Ownership / Safety |  |  |  |
| *Current Personal Firearm Ownership (*n*=518)* | 143 (40.97%) | 89 (52.66%) | 0.01 |
| *Household Firearm Ownership (*n*=516)* | 190 (54.60%) | 112 (66.67%) | 0.009 |
| *Feel Safer with Firearm in* *Home (*n*=512)* | 226 (65.32%) | 121 (72.89%) | 0.09 |
| Firearm Characteristics |  |  |  |
| Among Current Firearm Owners |  |  |  |
| *Number of Firearms Owned (*n*=221)* |  |  | 0.14 |
| One | 69 (50.00%) | 33 (39.76%) |  |
| Multiple | 69 (50.00%) | 50 (60.24%) |  |
| *Types of Firearms (*n*=228)* |  |  | 0.02 |
| Handguns only | 91 (64.54%) | 42 (48.28%) |  |
| Long guns only or both | 50 (35.46%) | 45 (51.72%) |  |
| *Primary Reasons for Firearm Ownership*^a^ *(*n*=232)* |  |  | 0.36 |
| Recreation | 22 (15.38%) | 16 (17.98%) |  |
| Protection | 104 (72.73%) | 56 (62.92%) |  |
| Recreation and protection | 13 (9.09%) | 14 (15.73%) |  |
| Other | 4 (2.80%) | 3 (3.37%) |  |
| *Feel Safer with Firearm in* *Home (*n*=231)* | 122 (85.92%) | 80 (89.89%) | 0.38 |
| *Firearm Storage Practices*^b^ *(n=225)* |  |  | 0.49 |
| Low risk | 49 (35.77%) | 26 (29.55%) |  |
| Intermediate risk | 49 (35.77%) | 31 (35.23%) |  |
| High risk | 39 (28.47%) | 31 (35.23%) |  |
| Among Household  Firearm Owners |  |  |  |
| *Firearm Storage Practices*^b^ *(*n*=295)* |  |  | 0.26 |
| Low risk | 69 (37.50%) | 34 (30.63%) |  |
| Intermediate risk | 69 (37.50%) | 40 (36.04%) |  |
| High risk | 46 (25.00%) | 37 (33.33%) |  |

*Note.* Urban = medium-sized town, suburb, city. Rural = small town, rural. N values for each characteristic represent the total number of respondents who provided a response to that item and may be fewer than the total sample included. T-test or chi-square ran to examine differences between urban and rural women Veterans.

^a^Fisher’s Exact Test.

^b^Low risk was operationalized as locked and unloaded; intermediate risk as unlocked and unloaded or locked and loaded; and high risk as loaded and unlocked.

**Supplemental Table 3**

*Unadjusted and Adjusted Poisson Regression Models for Firearm Ownership and Characteristics by Rurality* *– Sensitivity Analyses with Alternate Conceptualization of Rurality*

|  | Unadjusted Models | | | Adjusted Models^a^ | | |
| --- | --- | --- | --- | --- | --- | --- |
| Model | *n* | PR (95% CI) | *p* | *n* | PR (95% CI) | *p* |
| **Full Sample (*N*=525)** |  |  |  |  |  |  |
| Current personal firearm ownership (current ownership vs no current/past ownership) | 518 | 1.29 (1.06, 1.56) | 0.01 | 516 | 1.32 (1.09, 1.60) | 0.005 |
| Household firearm ownership | 516 | 1.22 (1.06, 1.41) | 0.006 | 514 | 1.21 (1.04, 1.40) | 0.01 |
| Perceived sense of safety with firearm in home | 512 | 1.12 (0.99, 1.26) | 0.07 | 509 | 1.09 (0.97, 1.24) | 0.16 |
| **Current Personal Firearm Owners (*n*=232)** |  |  |  |  |  |  |
| Number of firearms (1 vs multiple) | 221 | 1.20 (0.95, 1.53) | 0.13 | 221 | 1.26 (0.98, 1.62) | 0.07 |
| Types of firearms (handguns vs long guns/both) | 228 | 1.46 (1.08, 1.97) | 0.01 | 227 | 1.42 (1.04, 1.93) | 0.03 |
| Perceived sense of safety with firearm in home     (yes/no) | 231 | 1.05 (0.95, 1.15) | 0.36 | 229 | 1.03 (0.93, 1.14) | 0.54 |
| Firearm storage risk^b^ (low/intermediate vs high) | 225 | 1.24 (0.84, 1.82) | 0.28 | 224 | 1.26 (0.85, 1.87) | 0.25 |
| Firearm storage risk^b^ (low vs intermediate/high) | 225 | 1.10 (0.91, 1.32) | 0.33 | 224 | 1.09 (0.90, 1.32) | 0.37 |
| **Household Firearm Owners (*n*=302)** |  |  |  |  |  |  |
| Firearm storage risk^b^ (low/intermediate vs high) | 295 | 1.33 (0.93, 1.92) | 0.12 | 294 | 1.39 (0.96, 2.01) | 0.08 |
| Firearm storage risk^b^ (low vs intermediate/high) | 295 | 1.11 (0.94, 1.31) | 0.22 | 294 | 1.12 (0.95, 1.33) | 0.19 |

*Note*. PR = prevalence ratio; CI = confidence interval. N values for each characteristic represent the total number of respondents who provided a response to that item and thus included in that analysis. Urban = medium-sized town, suburb, city. Rural = small town, rural. Reference group = urban residing women Veterans. The independent variable was rurality (rural vs urban); dependent variables (outcomes) are listed in the lefthand column.

^a^Adjusted for education, race (Black vs all other races), and sexual orientation (heterosexual vs all other).

^b^Low risk was operationalized as locked and unloaded; intermediate risk as unlocked and unloaded or locked and loaded; and high risk as loaded and unlocked.
